# Embedding Responses in Spontaneous Neural Activity Shaped through Sequential Learning

**DOI:** 10.1371/journal.pcbi.1002943

**Published:** 2013-03-07

**Authors:** Tomoki Kurikawa, Kunihiko Kaneko

**Affiliations:** 1 Graduate School of Arts and Sciences, University of Tokyo, 3-8-1 Komaba, Meguro-ku, Tokyo, Japan; 2 Research Center for Complex Systems Biology, University of Tokyo, 3-8-1 Komaba, Meguro-ku, Tokyo, Japan; University of Oxford, United Kingdom

## Abstract

Recent experimental measurements have demonstrated that spontaneous neural activity in the absence of explicit external stimuli has remarkable spatiotemporal structure. This spontaneous activity has also been shown to play a key role in the response to external stimuli. To better understand this role, we proposed a viewpoint, “memories-as-bifurcations,” that differs from the traditional “memories-as-attractors” viewpoint. Memory recall from the memories-as-bifurcations viewpoint occurs when the spontaneous neural activity is changed to an appropriate output activity upon application of an input, known as a bifurcation in dynamical systems theory, wherein the input modifies the flow structure of the neural dynamics. Learning, then, is a process that helps create neural dynamical systems such that a target output pattern is generated as an attractor upon a given input. Based on this novel viewpoint, we introduce in this paper an associative memory model with a sequential learning process. Using a simple Hebbian-type learning, the model is able to memorize a large number of input/output mappings. The neural dynamics shaped through the learning exhibit different bifurcations to make the requested targets stable upon an increase in the input, and the neural activity in the absence of input shows chaotic dynamics with occasional approaches to the memorized target patterns. These results suggest that these dynamics facilitate the bifurcations to each target attractor upon application of the corresponding input, which thus increases the capacity for learning. This theoretical finding about the behavior of the spontaneous neural activity is consistent with recent experimental observations in which the neural activity without stimuli wanders among patterns evoked by previously applied signals. In addition, the neural networks shaped by learning properly reflect the correlations of input and target-output patterns in a similar manner to those designed in our previous study.

## Author Summary

The neural activity without explicit stimuli shows highly structured patterns in space and time, known as spontaneous activity. This spontaneous activity plays a key role in the behavior of the response to external stimuli generated by the interplay between the spontaneous activity and external input. Studying this interplay and how it is shaped by learning is an essential step toward understanding the principles of neural processing. To address this, we proposed a novel viewpoint, memories-as-bifurcations, in which the appropriate changes in the activity upon the input are embedded through learning. Based on this viewpoint, we introduce here an associative memory model with sequential learning by a simple Hebbian-type rule. In spite of its simplicity, the model memorizes the input/output mappings successively, as long as the input is sufficiently large and the synaptic change is slow. The spontaneous neural activity shaped after learning is shown to itinerate over the memorized targets in remarkable agreement with the experimental reports. These dynamics may prepare and facilitate to generate the learned response to the input. Our results suggest that this is the possible functional role of the spontaneous neural activity, while the uncovered network structure inspires a design principle for the memories-as-bifurcations.

## Introduction

The way in which neural processing of sensory inputs leads to cognitive functions is one of the most important issues in neuroscience. Neural activity in the presence of sensory stimuli [1]–[4] and during the execution of cognitive tasks in response to sensory inputs have been measured experimentally [5], [6], and neural network models that exhibit the requested responses to the inputs have been investigated theoretically [7]–[12]. Learning algorithms have also been proposed to memorize several input/output (I/O) mappings [12]–[16].

The response activity has been the main focus both in modeling studies and experiments, while pre-stimulus, i.e., spontaneous, activity has been dismissed simply as background noise. However, spontaneous activity has recently been garnering more attention since experimental measurements have revealed that the spontaneous activity is not random noise and that it shows characteristic spatiotemporal patterns [17]–[19]. Furthermore, many observations have revealed that the response activities to external stimuli [20], [21] or cognitive tasks depend on the spontaneous activity [22], [23]. Evoked responses are generated not only by external inputs but also through the interplay of the spontaneous activity and external stimuli. Thus, to establish a neural basis for the cognition and computation in a neural system, it is important to understand the nature of this interplay.

Spontaneous activity has been analyzed theoretically over the last few decades by using neural network models of rate-coding or spiking neurons with random, designed, or biologically realistic connections [24]–[28]. However, apart from a few publications [29], [30], the relationship between the spontaneous activity and response to external input has rarely been investigated. Furthermore, how the learning shapes the spontaneous activity and its response to an input is still an open question, but recent experimental studies suggest that learning and developmental processes modify and shape the spontaneous activity [31], [32]. In the present paper, we analyze how the spontaneous activity is formed when I/O mappings are memorized. We do this by introducing a simple learning rule to the neural dynamics in order to study the interplay between the spontaneous activity and input-evoked response.

To analyze the formation of the spontaneous activity and its response to the memorized input through the learning of I/O mappings, we previously proposed a novel view on memory in [33], [34], which we called “memories as bifurcations” in contrast to the traditional theoretical viewpoint of “memories as attractors.” According to the memories-as-attractors viewpoint, each memory is embedded in one of the attractors in a unique neural dynamical system [11]. An input specifies an initial condition of the dynamical system, and from that initial state, the neural activity reaches an attractor that matches the target corresponding to the given input. Thus, the initial states are determined by the given inputs, but the neural activity in the absence of inputs is not examined. In contrast, according to the memories-as-bifurcations viewpoint, an input modifies the neural dynamics as a parameter, and the flow structure of the neural activity is also changed from that without an input. In the absence of input, the neural activity evolves and corresponds to spontaneous activity. In the presence of a learned input, the flow structure in the neural dynamics changes and an attractor that matches the requested target corresponding to the applied input emerges. With an increase in the input strength, the flow structure changes via a sequence of bifurcations in terms of dynamical systems theory. Here, the flow structure can be changed substantially by applying different memorized inputs. Thus, for this viewpoint, memories are embedded in the flow structure of the neural dynamics such that they enable appropriate bifurcations to appear upon input application.

Previously, we designed a neural-network connection matrix through correlations among memorized inputs and targets so that an output that matches a target is generated, as a result of bifurcations from the spontaneous activity, by applying the corresponding input [34]. In the model, similarity between the spontaneous and evoked activities was demonstrated and is consistent with recent observations in experimental studies [32], [35]–[37]. Although the simplicity of the model is an advantage for analyzing the relationship between spontaneous and evoked neural activities, it remains unclear whether the simplistic structure in the designed network in [34] is the only way to store associative memories or if there exists a variety of networks that show similar behavior and generate a sufficient memory capacity. Also, how such network structures for memorizing I/O mappings are formed by learning through a widely-accepted synaptic plasticity rule, such as the Hebbian rule, is still open for debate.

In the present study, we introduce a sequential learning model with a simple Hebbian-type learning rule that changes the synaptic strength according to the activities of the pre- and postsynaptic neurons. From extensive numerical simulations, we have confirmed that through this learning the networks memorize 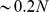 mappings (where 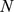 is the number of elements) satisfying the memories-as-bifurcations viewpoint. Here, spontaneous activity shows chaotic behavior with approaches to memorized output patterns. By applying each memorized input, this activity is transformed (after a sequence of bifurcations) into different attractors that generate the target pattern corresponding to the applied input.

In spite of the sequential learning scheme, the neural network does not lose the memory it learned earlier; it has a capacity of up to 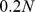. This capacity is not so small, and interestingly it is not possible in conventional sequential learning models in which the learning of a new I/O mapping easily pushes out previous memories. As long as the memorized targets are attractors in the same dynamical system, the formation of a new attraction to a novel attractor will easily destroy the attraction to earlier target patterns. Our model differs in that the different targets are attractors in the presence of the corresponding input, i.e., they are embedded in different neural dynamical systems, so that attractors for earlier targets are not destroyed. Here, the spontaneous activity is flexible; it is possible to apply an input so that a new target is embedded in the network structure without destroying the information of the previous targets.

Remarkably, the network generated through the learning process to obtain a high memory capacity is found to have a similar structure to the network designed in [18]. Although the learning process can generate a huge variety of networks, which are not similar to the designed network, a common structure is generated by the learning. A simple learning rule for synaptic change is sufficient for generating such a network.

## Model

We consider a system composed of 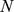 continuous rate-coding neurons whose activity 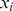 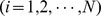 lies between −1 and 1 and evolves according to
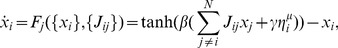
(1)where 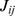 denotes a connection from the 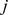-th to 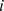-th neuron, 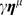 is an input pattern 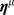 with input strength 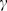 and 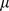 is index of learned mappings. 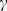 can represent the strength of sensory input, for example, the contrast of visual stimulus and the concentration of odorant.

For each input pattern 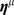, we set a pattern 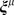 as the target, and the input and target patterns are generated as random 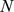-bit binary patterns, with probabilities 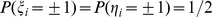. We postulate that by applying each input pattern 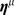, the corresponding target pattern 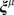 is recalled, i.e., an attractor matching the target 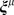 is generated. We adopt the following learning procedure to embed the postulated I/O mappings.

### Learning procedure

We first select two random binary patterns, 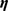 and 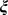, as the input and target patterns, respectively. The neural activity evolves in the presence of 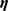 whose strength 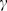 is constant during the learning process for 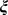. The synaptic connection 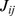 also evolves according to
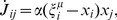
(2)where 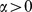 is a learning parameter that is the inverse of the time scale ratio of the synaptic to neural dynamics. The above synaptic dynamics are determined by correlations between the activities of the pre- and postsynaptic neurons. This learning rule takes a similar form as the perceptron learning rule where the synaptic connection is changed by correlations between activities of elements in the input and output layers [16].

Here, although the validity of this learning rule is not mathematically proven in contrast to the perceptron, it is expected by the following argument. According to Eq. 1, the change in the neural activity during 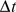 with the connection modified by the learning, 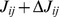, is given by
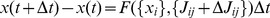
(3)

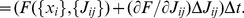
(4)Following the synaptic dynamics in Eq. 2, the change in the neural activity due to 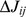 is given by
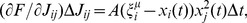
(5)where 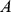 is a positive value determined by 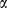 and differential coefficient. Thus, when 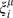 is larger (smaller) than 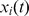, 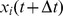 increases (decreases), respectively. Hence, the change in the synapses will drive the successive activity toward the target 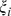. Note, however, that the distance between the neural activity and the target is not necessarily guaranteed to decrease monotonically through the learning, because the total change in the neural activity 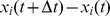 depends also on 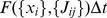.

The learning process stops automatically when the neural activity matches the target since in this case 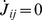, otherwise, the learning process continues. Here we impose several I/O mappings to be successively learned, and after learning the preceding mapping, another input pattern with the same strength as the previous learning is applied while giving a new target pattern. The learning process for each single I/O mapping is called a learning step in what follows. In this learning algorithm, which belongs to a class of palimpsest learning models [38]–[40], each mapping is learned sequentially and previously learned mappings are overwritten by the latest mapping. Thus, it is possible that older mappings are forgotten through the learning process.

During the learning process, double (neural and synaptic) dynamics run concurrently, and the neural and synaptic states have to be set as initial states: the neural and synaptic states are randomly selected from 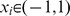 with a uniform probability and from a binary ensemble of 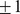 with equal probability, respectively. In this model, fully-connected networks without self-connections are used. Through different learning processes, different sets of mappings are learned so that the generated networks are also different. For a statistical analysis, we take an average over many networks shaped through different learning processes.

As our purpose in this study is to analyze the relationship between the spontaneous and evoked dynamics, we analyze the neural dynamical system in the absence and presence of input after learning. After the learning is completed, the synaptic connections are fixed and only the neural activities evolve. Note that there is no need for the input strengths for learning and memory recall to be identical: we can set the input strength 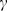 used during the recall process after the learning and independently of the input strength used during the learning process. For example, after learning with 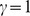, we can analyze the evoked dynamics by applying the input with 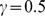. To distinguish the two clearly, the input strength used in the learning process is denoted by 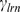 and that used in the analysis of the neural activities after learning is denoted by 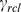. The spontaneous and evoked dynamics are given by 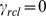 and 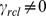, respectively.

### Definition of memory

As recall and memory for the memories-as-bifurcations viewpoint are defined differently from those for the memories-as-attractors viewpoint, we outline the definitions of recall and then memory here. A network succeeds in recalling a target 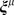 for an input of 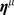, if, on application of input 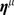 for 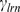 = 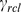, the overlap of the evoked activity with the target 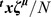 is higher than the overlap with any other pattern 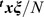. Here, 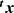 is a transposed vector of 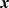 and the inner product 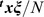 is given by 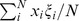. By considering a case in which the evoked attractor is not a fixed-point attractor, the temporal average overlap is taken as this criterion. By denoting the temporal average overlap with the target 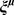 as 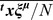, the criterion for the successful recall of 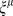 corresponding to the applied input 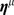 is given by
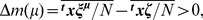
(6)where we measure the avaraged overlaps in the presence of the input 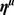 and 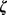 is the pattern that has the largest overlap with the activity among other targets and inputs, as well as other random patterns.

Memory is defined as the ability of a network to recall a target for most initial states. The condition for whether a network memorizes an I/O (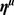/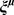) mapping is
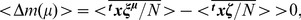
(7)where 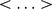 represents the average over the initial states of this network. By extending this criterion, we adopt a condition for determining whether networks memorize the I/O mapping for a certain parameter as
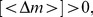
(8)where 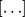 denotes the average over different networks.

## Results

To examine whether a network shaped through the learning process memorizes the I/O mapping(s), we measure the evoked activity. Then we analyze the possible relationship between the spontaneous and evoked activities, and also analyze the characteristic features of the connection matrix 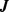 that allows for memory.

Due to the high dimensionality of neural dynamics, it is difficult to directly analyze the time evolution in the entire phase space. Instead, we mainly use the overlaps of the neural activities with some patterns: that with the target 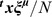, that with the input 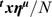, and that with a randomly selected pattern 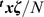. The behaviors of these overlaps are characteristics of the neural dynamics. We focus on the dependence of the neural and synaptic dynamics on two parameters: the learning parameter 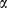 and the input strength 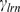. We begin by examining the dependences after one learning step of only one mapping and then examine the dependences after multiple learning steps.

### Neural dynamics formed through one learning step

**Figure 1:**
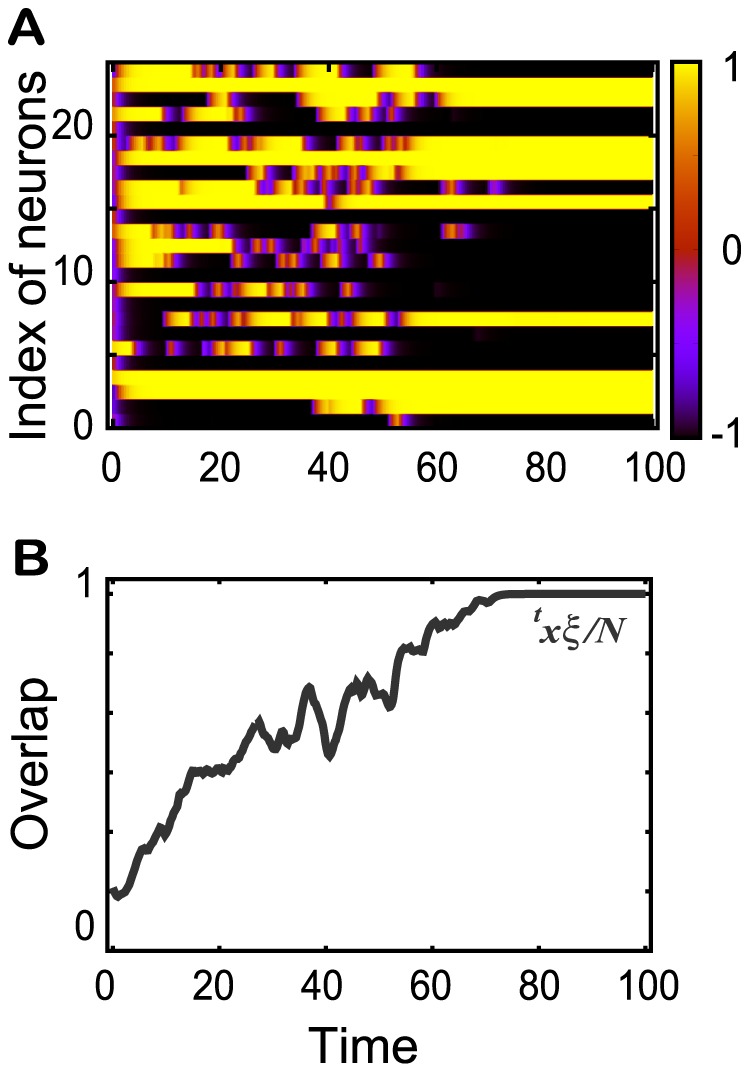
Learning process for one mapping. **A.** A raster plot of the activity 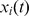 for 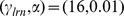 and for 25 of 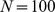 neurons. **B.** The temporal evolution of the overlap with the target 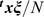 for the learning process in A.

Fig. 1 exhibits a learning process shown as a raster plot and the time series of the overlap with the target 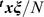 for 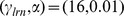. After wandering over many neural activity patterns, the neural activity reaches the target pattern and the learning process is completed. The learning process does not stop by becoming trapped in a local minimum, nor does it continue to wander over the neural patterns. We confirmed that in all trials with parameters of 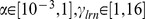, the learning was completed.

**Figure 2:**
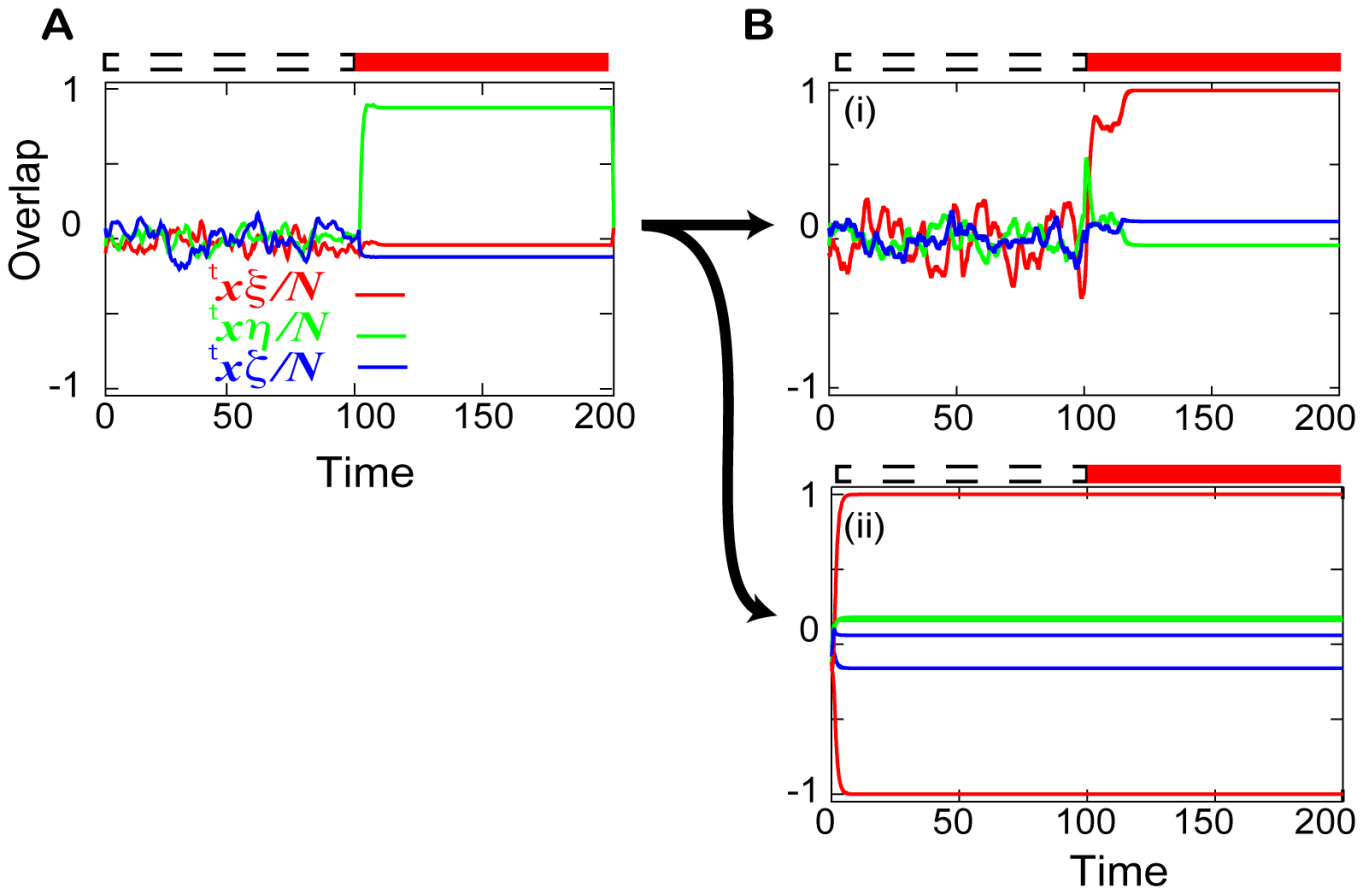
Recall processes before and after the learning. Neural activities plotted as a time series of the overlaps with the target (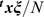), the input (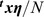), and a random pattern (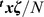). The random pattern is generated from the same ensemble of targets and inputs. **A.** The recall process before the learning for 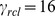. **B.** The recall processes after the learning for (i) 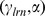 = (16,0.01) and (ii) 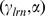 = (1,0.5). The activity is spontaneous (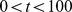) or evoked (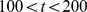) as indicated by the dotted and filled red bars, respectively, above the plots. The evoked activity is introduced by the application of an input of strength 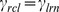. In (ii), the time series from two initial conditions that lead to the two different attractors are plotted.

During a learning process, the flow structures of the spontaneous and evoked activities change. Hence, the recall process also changes through the learning process. Fig. 2 shows a recall process before and after learning for 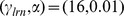 and 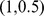. Before learning, an attractor matching the applied input pattern is generated when that input is applied (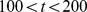 in Fig. 2A), but the overlap with the required target is not high and the network thus fails to recall the target. After learning, two types of neural dynamics are generated depending on the parameter values (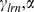) (see also Table 1):

**Table 1:** Characteristics of each regime.

Regime	Response (R)	Non-response (NR)
Spontaneous activity	Chaotic behavior wandering among targets	Fixed points that match the target and reverse target
Evoked activity	Target fixed point	No change from the spontaneous activity
Capacity	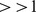 ( 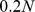 )	0 or 1
Network structure	Asymmetric based on input/output correlations [34]	Mattis type

- The spontaneous activity shows chaotic behavior around the origin, while the evoked activity shows stationary activity, which matches the target pattern (shown in Fig. 2B(i)), and the neural activity responds to the applied input. This regime is referred to as the “response” (R) regime.
- Only fixed-point attractors that match the target and the “reverse” target patterns exist both in the absence and presence of the input (shown in Fig. 2B(ii)). Here, the reverse target pattern represents a neural pattern in which all the variables take the opposite sign of those of the target pattern. The neural activity in this case does not respond to the input, and the regime is referred to as the “non-response” (NR) regime.

**Figure 3:**
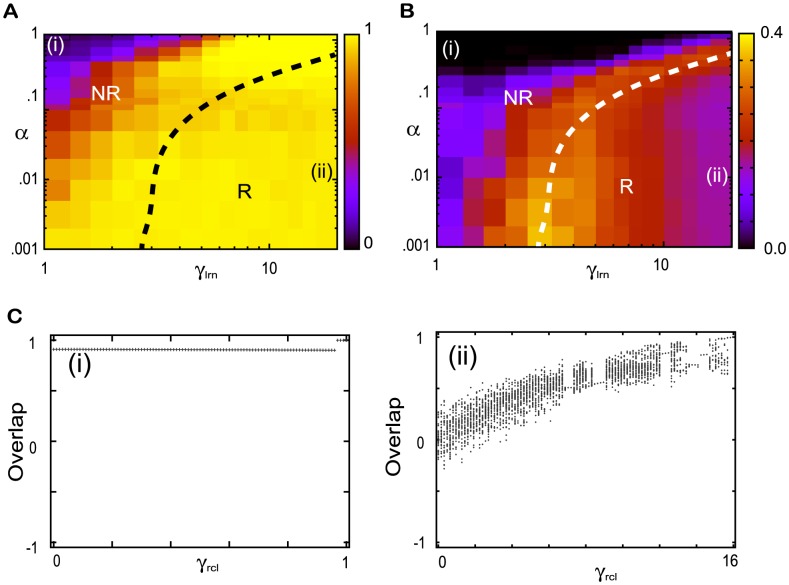
Phase diagram of the evoked and spontaneous dynamics and bifurcation diagram. **A.** The quenched average of the overlap with the target 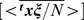 in the evoked dynamics. **B.** The standard deviation (SD) of the overlap averaged over time and over the networks 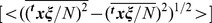. Average values in A and B are computed over 100 networks and over 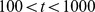. The dotted curves in A and B, plotted for reference, show the boundary between the R and NR regimes and, which are computed by the ridge of SD in B with smoothing the line. **C.** The local maxima in the 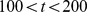 time series of the overlap with the target 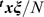 as a function of the input strength in (i) the NR regime for 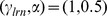 and (ii) the R regime 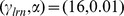 showing the bifurcations.

We now analyze the spontaneous and evoked neural dynamics in these two regimes. First, to reveal the dependence of the evoked dynamics on the parameters, 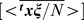 as a function of 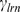 and 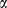, is shown in Fig. 3A. In the R regime, for larger 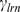 and smaller 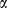 values, only the target attractor exists and the average overlap is equal to one, while in the NR regime, both the target and reverse-target attractors exist and the average overlap is lower than that in the R regime. As 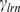 decreases or 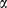 increases, the volume of the reverse-target attractor basin increases and that of the target attractor decreases so that the average overlap with the target 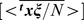 also decreases. The dotted line in Fig. 3A represents the boundary between the R and NR regimes computed using the spontaneous activity, as discussed below.

To analyze the spontaneous dynamics, we note that due to the symmetry, the mean overlap for each target over time is generally zero because the orbit can approach both the target and reverse-target with equal probability. Thus, we measure the standard deviation (SD) of the overlap to quantify the approach to each target. The SD(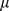) of an overlap with the 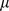-th target over time is computed as 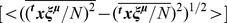. If this SD is much larger than that for the overlap with a random pattern, then the spontaneous activity selectively approaches the target (and its reverse). A numerical computation of the SD as a function of 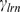 and 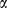 is plotted in Fig. 3B. In the R regime, chaotic behavior appears and the SD takes a finite positive value, while in the NR regime, fixed-point attractors exist and so the SD is zero. Interestingly, a band that has a higher SD, which stretches from (2.6, 0.001) to (16,1), and whose ridge divides the R and NR regimes appears in the figure. In Fig. 3B, the ridge is shown as the dotted line, which is also plotted as a reference in Fig. 3A.

Around the ridge, the SD of the spontaneous activity is much higher than that in other areas, and the chaotic spontaneous activity shows switching behavior between the target and reverse target. While the target and reverse-target attractors are unstable, their ruins still exist and the neural dynamics intermittently visit them.

In Figs. 3A and B, the boundary defined by the SD might be slightly ambiguous because of the finite-size effect. However, by extrapolating the result for larger system sizes (to be discussed later), it is expected that, in the absence of inputs, all the networks in the NR regime show fixed-point behavior and those in the R regime show chaotic behavior, in the thermodynamic limit. By increasing 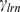 or decreasing 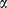, the minimum distance between the activity and the target (or the reverse-target) increases in the R regime. Thus, in this limit, the SD in the NR regime is zero. It suddenly increases to nearly one at the transition point, and then gradually decreases in the R regime. The ridge of the SD thus indicates the transition between the NR and R regimes well. The area with the average overlap taking nearly one above the dotted line in Fig. 3A is expected to remain even in the thermodynamic limit. However, this area is included in the NR regime, since according to the analysis of the neural dynamics after multiple learning steps, to be discussed later, no more than a single pattern is recalled, as in the rest of the NR regime.

We also show how spontaneous activity changes into evoked activity with an increase in 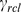 in each regime, as shown in Fig. 3C. In the R regime, by increasing 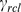 from zero, the neural activity shows successive bifurcations such that the overlap with the target 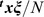 increases to approach unity at 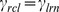. The fixed-point attractor matching the target appears at 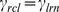. In the NR regime, the target and reverse target attractors do not change on application of the input, but the basin volumes of the attractors increase.

### Connection matrix shaped through the learning process

**Figure 4:**
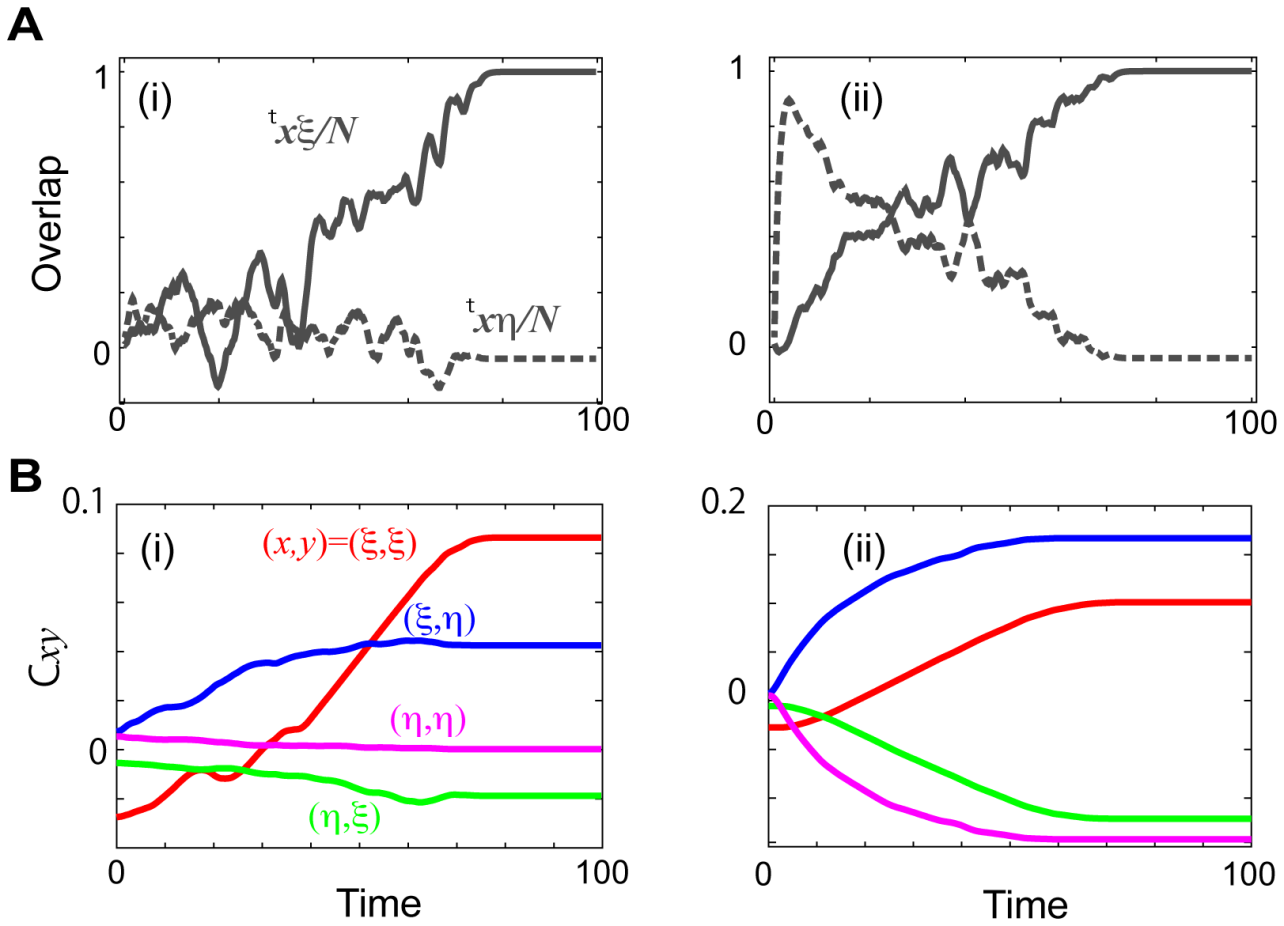
The time evolution of the overlap and the matrix elements. **A.** The overlaps with the target 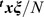 and input 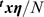 during the learning process (i) in the NR regime for 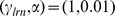 and (ii) in the R regime for 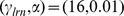. **B.** The matrix elements 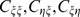, and 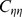 in (i) the NR regime and (ii) the R regime with the same parameters as in A.

We analyze the connection matrix that is shaped through the learning process, in the R and NR regimes, by measuring the element of the matrix C which is projected onto 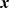 and 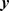, as defined by
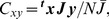
(9)where 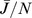 = 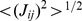. Note that for a given binary pattern 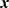, if the system has a large matrix element 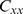, then pattern 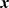 is more stable in the absence of inputs for the neural dynamics in Eq. (1). Similarly, when 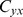 is larger, 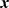 is less stable. Fig. 4 shows a time series of the elements 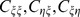, and 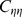 for the NR, 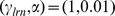 and R, 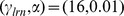 regimes. In the NR regime, only the 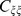 element is much larger than the others after learning, while in the R regime, both 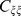 and 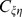 take salient positive values and 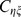 and 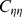 take salient negative values.

The result that 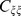 dominates in the NR regime means that the generated connection matrix takes a similar form to that of the Mattis model in a spin system [41], which corresponds to the Hopfield network with only one memorized pattern. In the network where 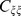 is larger and the other elements are much smaller, the target 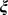 and reverse-target patterns 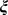 remain highly stable. This is consistent with the above analysis in the NR regime. In the R regime, in contrast, the connection matrix shows a form distinct from those of the matrices in Mattis and Hopfield-type networks. Remarkably, the matrix takes a similar form to that of the model in [34], where 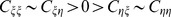 was adopted. Indeed, the behaviors of the spontaneous and evoked activities in this regime agree with that observed in that model [34].

**Figure 5:**
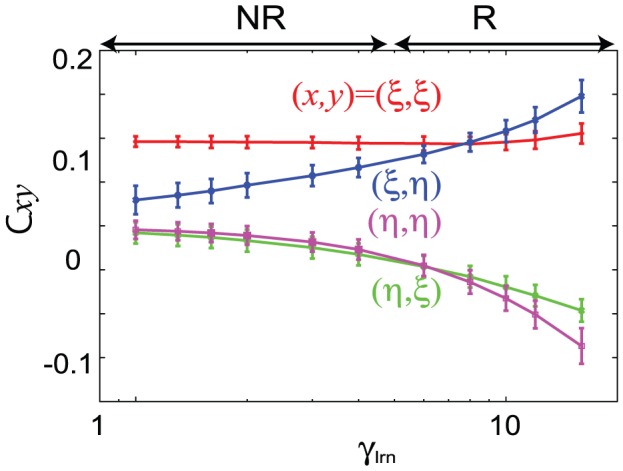
Dependence of the matrix elements 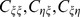, and 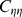 on the learning parameter 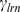. The matrix elements averaged over 100 networks for a fixed 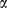 of 0.01 are shown, and the corresponding regimes (NR and R) are indicated above the figure. The error bars represent the standard deviation.

In general, the behaviors are strongly dependent on the matrix elements. In Fig. 5, the elements as a function of 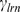 are plotted. For 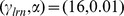, all of the elements deviate saliently from zero, and as 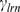 decreases, the elements, 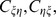 and 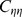 decrease rapidly, while 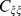 does not change. The regime changes from the R to NR regime as this occurs.

We now analyze why such connection matrices are formed through the learning process. The evolution of the matrix element 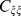 is also determined by Eq. (2) as follows:
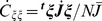
(10)

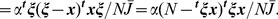
(11)Although 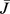 also evolves temporally, we set 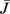 as a constant value, because relative scale of the elements is relevant for understanding the behavior. In the same way, the evolutions of the other elements are determined by
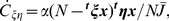
(12)

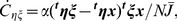
(13)

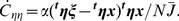
(14)In both the regimes, the activity 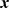 approaches a target 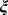 and thus 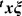 is greater than zero (and smaller than 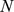) for most of the learning process. Thus, 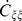 is positive for most of the learning process and then, 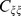 takes a large positive value. In contrast, the change in the other elements is distinct between both regimes, which is explained by the initial behavior of the learning process. In the R regime, the overlap with the input 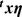 increases in the early stage of the learning process as 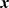 is directed toward 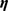 by the input, as shown in Fig. 4A(ii). It is estimated that 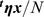 is 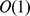 and positive, which is much larger than 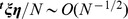. Thus, 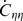 and 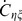 are negative in the R regime, while 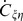 is positive. These estimates of the sign of the elements are consistent with the matrix elements in Fig. 4B. For the NR regime, in which 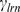 is smaller and 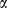 is larger, the increase in the overlap with the input 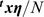 in the early stage is much smaller than that in the R regime; if 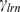 is small, the neural activity does not respond strongly to the input, whereas if 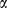 is large, the learning is completed before the overlap with the input increases. Thus, the temporal changes in 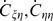, and 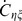 are much smaller. Hence, only 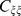 takes a large value, and thus the Mattis-type network is generated.

### Neural dynamics formed through multiple learning steps

**Figure 6:**
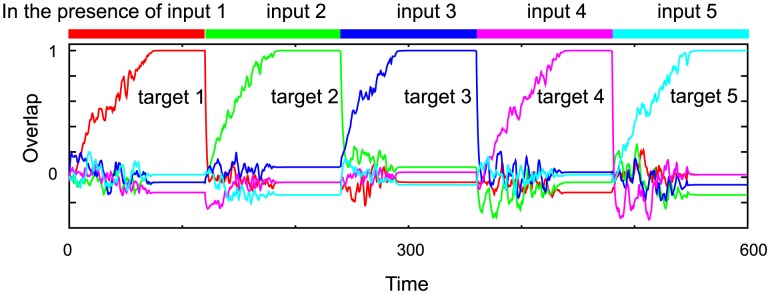
A learning process for five mappings. The time evolutions of 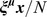 (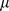 = 1, 2, 3, 4, and 5) are indicated by different colors for 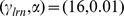. In the presence of each input (shown as the colored bar above the plot), the neural activity converges to the target to be learned. After convergence, a new mapping is provided, and in the presence of the new input, the system starts to learn the new target.

Neural activities that are shaped through multiple learning steps are analyzed for I/O mappings that are learned sequentially, as shown in Fig. 6. In the presence of each input (as indicated by the colored bars above the plot), the neural activity converges to the target to be memorized in the same way as in the learning process of a single mapping (shown in Fig. 1). Note that although the learning process changes the synaptic connections and flow structure of the neural activity, some of the structure generated in earlier learning steps is preserved because the change in the flow structure in each learning step occurs in the presence of a different input pattern. We mainly present the results after the learning of 40 mappings and analyze the behaviors of spontaneous and evoked activities for later 30 mappings in the following analysis. (We choose 30 mapping because memory capacity is almost 20 as shown later. The number 30 and 40 can be arbitrary, as long as they are chosen to be larger than the many capacity.)

Corresponding to each phase in the one-step learning, we also found two distinct behaviors in the multiple learning: (i) Neural activity responds to multiple inputs so that an attractor that matches each learned pattern is generated respectively upon each input. Thus, multiple mappings are successfully memorized. (ii) The neural activity does not respond to any input. The two attractors that match the latest learned target and its reverse pattern exist in the absence and presence of the input. Recall in response to an input is not observed either. We call these the R and NR regimes, respectively, in the same manner as the analysis for one-learning step.

**Figure 7:**
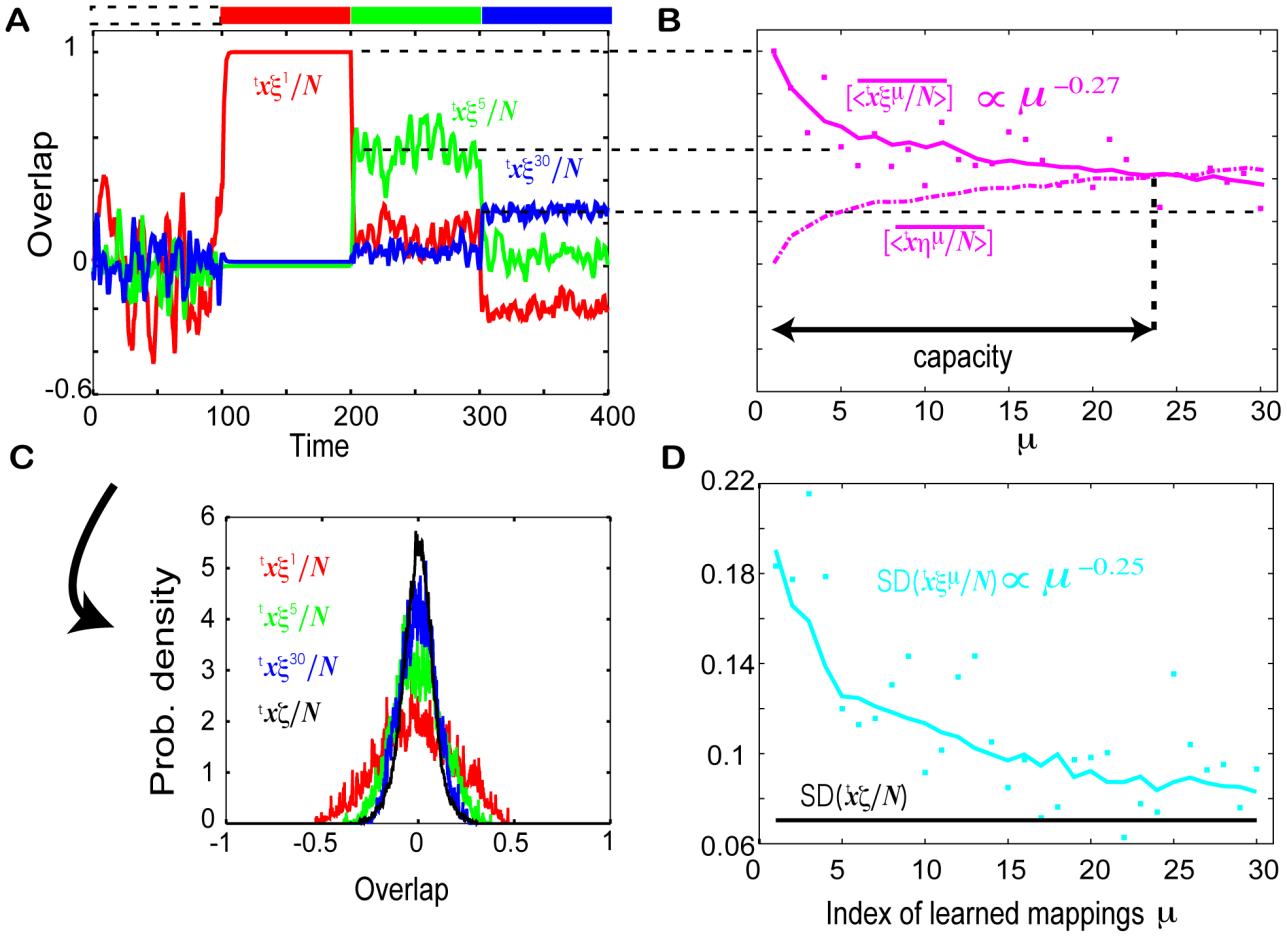
The neural dynamics after 40 learning steps in the response (R) regime. **A.** The time series of the neural activities shown by the overlap with the 1st, 5th, and 30th targets 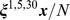 in the absence and presence of the 1st (red), 5th (green), and 30th (blue) inputs (shown by the colored bars above the plot) for 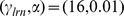. **B.** The time-averaged overlaps with the learned targets 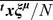 as a function of 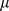 (squares). The overlaps with the targets and inputs averaged over the 100 networks are shown as the solid and dashed lines, respectively. **C.** The distributions of the overlaps of the spontaneous activity with the targets. The black line represents the distribution averaged over 10 overlaps with 10 random patterns as a control, and the others are distributions of the overlaps 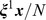, 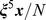, and 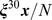 using the same colors as in A. **D.** The SD of the overlap with the target for the temporal evolution (squares), and the SD of the target and random pattern averaged over the 100 networks shown as the right blue and black lines, respectively.

In Fig. 7, we plot the neural dynamics in the presence and absence of inputs after 40 learning steps for 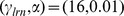 in the R regime. The recall processes of 1st, 5th, and 30th targets are shown by the overlaps with 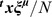 for 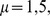 and 30 in the absence and presence of the 1st, 5th, and 30th input, respectively. From here on, the index 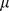 (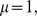 5, and 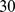 in this case) denotes the order of the I/O mapping beginning with the most recent, i.e., the 1st mapping is the latest learned one, while the 5th is that learned 5 steps earlier, and so forth.

In the R regime, by applying an input 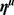, the overlap with the required target 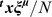 increases and takes on the highest value of all overlaps. In particular, in the presence of the latest input 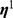, the overlap with the latest target 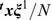 takes a much higher value, of nearly one, and an attractor that matches the latest target is generated. Thus, the latest target is successfully recalled by applying the corresponding input. In the presence of earlier inputs, the overlaps with the requested targets take smaller values than that with the latest target, but they are still larger than the overlaps with other patterns (see Fig. S1), as long as the retrieved mapping is not one that was learned much earlier (as shown below). (The overlaps with the applied inputs also take higher values than the overlaps with other patterns, as well as the overlaps with the required targets. Thus, we compare the overlaps with the targets with those with the inputs in the following part.) For example, the overlap with the 5th target 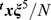 is highest among the overlaps with others, in particular higher than that with the 5th input 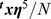 (Fig. 7B). Thus, the 5th target is also recalled according to Eq. 4. From almost all initial values, the neural activity evolves to an attractor that gives the corresponding target pattern upon application of the appropriate input. Thus, the 1st and 5th targets are always recalled. According to the definition of memory in Eq. 6, the 1st and 5th mappings are memorized in this network. In contrast, the overlap with the 30th target 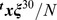, which is learned much earlier, takes a much smaller value and is lower than the overlap with the 30th input 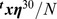. Thus the network cannot recall the 30th target, i.e., the target has not been memorized. Hence the memory capacity of the present network lies between 5 and 30.

To examine the memory capacity, we compute the average overlaps with the targets 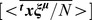 in the presence of each earlier input, as well as the average overlap with the input itself 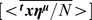, as shown in Fig. 7B. The overlap with an earlier target 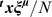 upon application of the corresponding input gradually decreases with an increase in 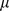, while the overlap with the applied input increases. The difference between the average overlaps with the 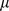-th target and input under the 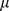-th input 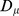 = 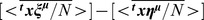 decreases with an increase in 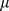. Here, 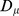 eventually crosses 0 at around 20. According to definition of memory in Eq. 8, the system in this regime succeeds in recalling the target by applying the corresponding input to 20 I/O mappings. To reduce the artifact from the fluctuations of the overlap on memory capacity due to the finite size effect, we modify the definition of the memory capacity 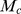 slightly as
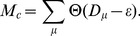
(15)Here, we set 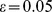, however, as long as the value is small, there is no essential change in the memory capacity. According to this modified definition, 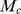 is computed to be 19.

We also analyze the spontaneous neural dynamics that underlie the responses to the learned inputs analyzed above in the R regime. The spontaneous neural activity shows noisy behavior, and no fixed pattern is stable, as shown in Fig. 7A. Irrespectively of the noisy behavior, the overlaps with the memorized targets often show high values from time to time. We compute the distributions over time of these overlaps and present them in Fig. 7C. The overlap distribution with the latest target 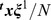 is much broader than that with a random pattern 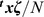, and thus, the neural activity gets selectively closer to the latest target from time to time, even in the absence of input. The distributions of the overlaps with earlier targets are also broader than that with a random pattern, even though the magnitude is smaller than that of the overlap with the latest target. Following the analysis introduced in the single-step learning, we measure the SDs of the distributions of the overlaps with all the targets, as represented by dots in Fig. 7D. We also compute the SD by averaging over the networks, as shown in Fig. 7D as the light blue line. As shown, the SDs of the later targets decrease as 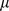 increases. The major source of decrease in the SD comes from a decrease in the amplitude of the overlap.

Therefore, the spontaneous activity approaches the learned targets from time to time and the closeness to the target 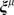 during the spontaneous dynamics decreases with 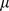. The SD decreases approximately as a power law as 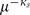, with 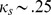. This decay rate roughly agrees with that of the evoked activity, which is approximated by 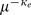 with 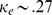. Both of the exponents are computed from a fit of the overlap and averaged SD to 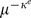 and 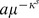, respectively, by using the least-squares method. We will analyze the dependence of the decay rates on the parameters 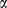 and 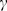 below.

In the NR regime, in contrast, the latest target and its reverse pattern exist as attractors in the absence and presence of inputs for 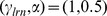 (see Fig. S2). This is identical to the NR-regime behavior after one learning step, for which 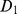 was nearly zero. Due to the stability of the latest target attractor, the neural activity does not respond to the earlier input 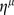 (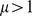) either, so that 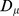 is also nearly zero. According to the definition of memory, Eq. 15, 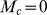. By decreasing 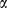 or increasing 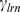, the reverse target attractor is less stable in the presence of the latest input, and loses stability at some parameter values, while this attractor is still stable in the absence of the input. In this region, 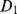 is equal to one, while there is still no response to an earlier input, and thus in this region, 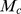 = 1.

### Bifurcation with an increase in the input strength

**Figure 8:**
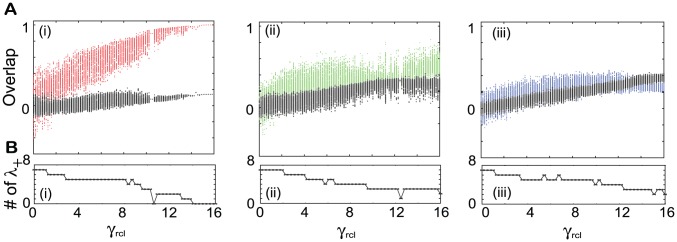
Bifurcation diagram for ( 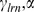
 **) = (16,0.01) in the R regime.** We use the network shaped after 40 learning steps. **A.** The local maxima in the 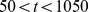 time series of the overlap with the target 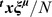 in the presence of the corresponding input 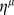 as a function of 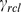. The overlaps with (i) the 1st (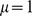), (ii) 5th (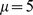), and (iii) 30th (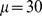) targets are plotted in red, green, and blue, respectively, while the data in black represent the overlap with each input 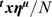 (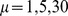). **B.** The number of positive Lyapunov exponents of these evoked dynamics as a function of 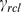. Lyapunov exponents are calculated from the time series 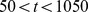 according to the algorithm in [56].

So far, we have analyzed the spontaneous neural activity with 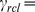
0 and the evoked activity with 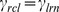. We now examine how the spontaneous activity is transformed into the evoked activity with 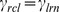, as 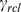 is increased. This change with changing 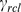 is regarded as a bifurcation or a sequence of bifurcations in terms of the dynamical system theory. The bifurcations of the neural activity, revealed by increasing 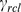 for the 1st, 5th, and 30th input strengths for the network given in Fig. 7, are shown in Fig. 8.

In the R regime, the overlap with the 1st (i.e., latest) target 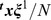 increases monotonically and continuously by increasing the strength of the 1st input. Finally, the fixed point that matches the 1st target is generated for not only the network used in the figure, but also most of the networks in the R regime. The change to a fixed point is understood as a low-dimensional bifurcation, while the whole sequence of neural activity changes involves higher-dimensional dynamics. For the 5th and 30th inputs, the overlap with the corresponding input is increased continuously with an increase in the input strength, in a similar manner as the bifurcation diagram for the 1st input. In contrast to the latest input, however, the attractor is not a fixed-point attractor even for 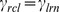, where the evoked activity still shows chaotic behavior.

Apart from the change to a fixed-point attractor, the bifurcation sequences involve a large degree of freedom in a high-dimensional (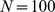) space. Hence, plotting a few macroscopic variables, i.e., the overlaps of the neural activity with a few targets, is not sufficient to capture the entire bifurcation sequence. Therefore, to consider the chaotic dynamics, we measured the Lyapunov spectrum for the neural activity dynamics. With an increase in the input strength, the number of positive Lyapunov exponents decreases, implying the existence of successive bifurcations from a high-dimensional attractor to a lower-dimensional attractor (see Fig. 8). Accordingly, the dimension of the neural-activity attractor also decreases. No positive Lyapunov exponents exist once the fixed-point attractor is reached for the input that was just learned, while even for the application of an earlier input, a decrease in the number of positive exponents is observed but the number does not reach zero.

In the NR regime, the latest target and reverse-target fixed-point attractors exist with 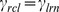. Even by increasing the input strength, these attractors remain stable and no bifurcation occurs.

### Dependence of the learned neural activities on the input strength and learning parameters

**Figure 9:**
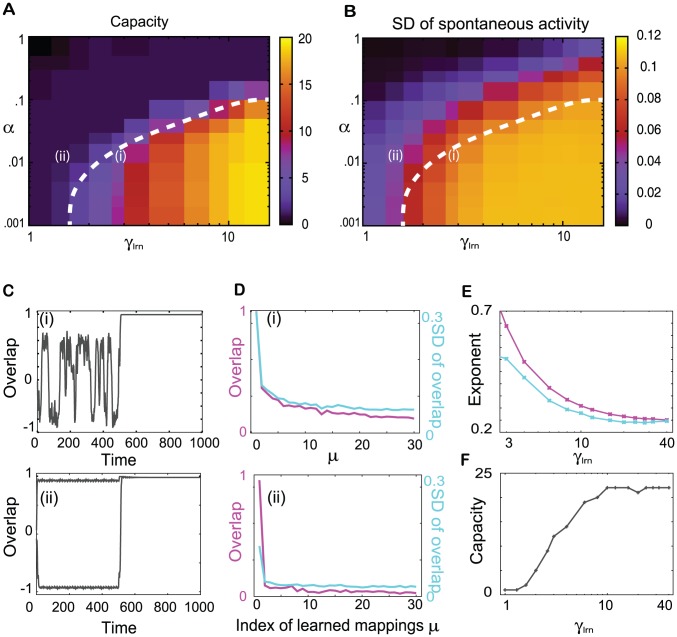
Dependence of the evoked and spontaneous activities on 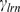
 **and** 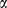 **.** **A.** The capacity (as defined in the main text). The dotted line denotes the boundary of the R regime, computed by the line where the memory capacity goes beyond one, with smoothing the line. **B.** The average SD of the spontaneous activity. In A and B, we computed the capacity and SD by averaging over 100 network and 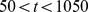. **C.** The temporal evolution of the overlap with the latest target 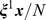 in the absence (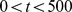) and presence (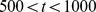) of the latest input with 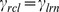 for 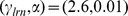 in (i) and for 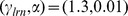 in (ii), indicated by (i), and (ii), for in A and B. For (ii), results from two initial conditions that lead to differed attractors are plotted. **D.** The average of the overlap with the 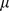-th target in the presence of the 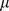-th input (magenta line) 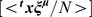 and the SD of the spontaneous overlap (right blue line) plotted as a function of 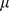 for the parameter set indicated by (i) and (ii) in A and B. **E.** The exponents 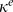 and 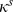, computed from a fit of the overlap and averaged SD to 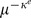 and 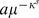, respectively. Both 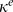 and 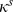 are computed for different 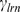 by fixing 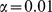 as represented by the magenta and right blue lines, respectively. **F.** The capacity 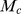 for different 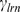 by fixing 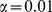.

The dependence of the spontaneous and evoked activities on the two parameters, 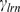 and 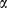, are analyzed through the capacity and SD. The dependence of the evoked activity is explored by measuring the capacity 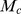 according to Eq. 15, with the results shown in Fig. 9A. In the R regime with a larger 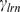 and smaller 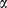, a high capacity is observed, while in the NR regime with a smaller 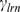 and larger 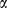, the capacity is zero or one. Over the entire parameter space, the overlap with the requested target in the presence of an earlier input decreases, i.e., 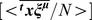 decreases as 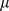 increases, while that with the corresponding input increases. However, the decay rate of the overlap with the target as a function of 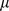 and the growth rate of the overlap with the input are dependent on 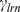 and 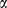.

For a large 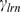 and small 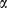, e.g., 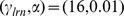 as shown in Fig. 7B, the decay rate of the overlap with the target as a function of 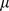 is small, as well as the growth rate of the overlap with the input. In general, when the capacity is higher, response to an earlier input is higher and the decay rates are lower. As the parameters approach the NR regime and the memory capacity decreases with a decrease in 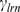 and increase in 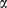, these rates become larger (see Figs. 7B and 9D(i)). Finally, in the NR regime, the rates reach maximal value, and the network responds only to the most recently learned input and not to any other input, i.e. 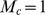 (see Fig. 9D(ii)).

To explore the dependence of the spontaneous activity, we measure the average SD of the spontaneous activity over the learned mappings,
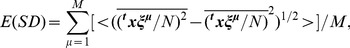
(16)as shown in Fig. 9B, where 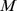 is set to 30. When 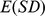 is larger, the decay rate of SD(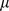) is smaller.

For a large 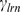 and small 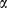, 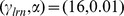, where 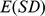 takes a higher value, the spontaneous activity approaches not only the latest target, but also an earlier target from time to time, as shown in Fig. 7D. The closeness to the target, as seen by the decrease in the SD of the overlap with an earlier target, decreases for targets memorized earlier. As 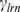 decreases and 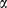 increases, and the system approaches the NR regime, the average SD decreases and this decay rate increases; the spontaneous activity approaches the latest target selectively as shown by the small distance between the spontaneous activity and the latest target (see Fig. 9C(i)). Finally, in the NR regime, the activity in the absence of input falls on the latest target and reverse-target pattern (or the localized fluctuations around these patterns) (see Fig. 9C(ii)).

The decay rates of the overlap with the evoked activity and the SD of the spontaneous activity in the R regime were seen to obey power laws of 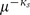 and 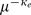, respectively, and the two exponents 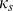 and 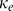 have a similar value for and dependence on 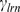, as shown in Fig. 9E. This suggests that the approach of the spontaneous activity to the target is correlated with the activity evoked in response to the corresponding input.

Both of the two exponents decrease for a larger 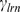 and smaller 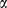. For much larger 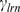 and much smaller 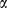 values, these decreases become saturated, and the curves of 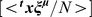 and SD(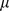) as functions of 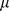 no longer change with an increase in 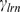. Thus, the capacities for different 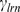 values become also saturated and take a common value of 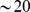 (Fig. 9F). In other words, for a sufficiently large 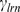 and small 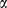, the capacity in this model with 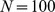 takes a constant value of 20. Further, from results for 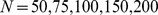, and 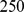, we have confirmed that this capacity is proportional to 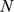; the capacity has a universal limit of 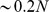 (see Fig. S3).

Note that the R and NR regimes are clearly distinguishable mathematically. Although the boundary between them might slightly ambiguous, as seen in Figs. 9A and B for 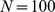 because of the finite-size effect, it is clearer with the increase in 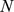, and, in the thermodynamic limit, it is expected that the memory capacity is equal to one (or zero) as is the fixed-point spontaneous activity, i.e., 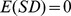, for all networks in the NR regime. In the R regime, in contrast, spontaneous activity shows chaotic behavior, i.e., 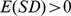, for all networks, and the memory capacity increases linearly with size 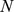, as 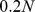. The proportion coefficient 0.2 may be slightly varied according to the criterion for the memory capacity, but the proportionality to 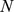 is invariant. Hence, the boundary between R and NR is clearly defined.

### Connection matrix shaped through multiple learning steps

**Figure 10:**
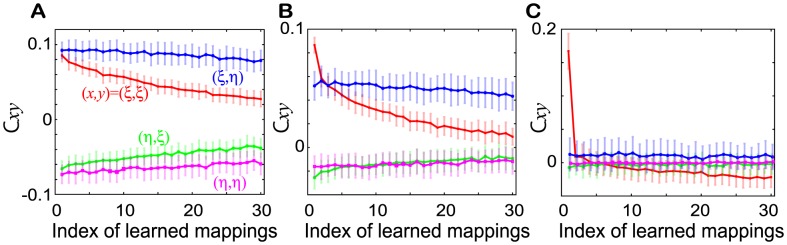
The matrix elements in the presence of the targets 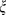
 **and inputs** 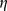 **.** The matrix elements 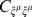, 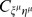, 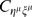, and 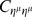 plotted as functions of 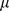 for **A**. 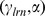 = (16, 0.01) in the R regime, **B**. 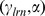 = (2.6, 0.01) in the boundary regime, and **C**. 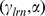 = (1,0.5) in the NR regime. The same colors as those used in Fig. 5 are used here. The error bars represent the standard deviation.

Finally, we analyze the connection matrix by measuring the elements of the matrix 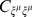, 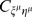, 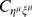 and 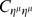 as defined in Eq. 9. In Fig. 10, we show the elements in both the R and NR regimes and also in the border between them. The elements in the R regime take comparable values for each 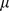, and decrease with an increase in 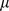, but the decay rates are rather small compared with those in the NR regime. Thus, for each mapping, the analysis of the network structure in the R regime after a single learning step is also valid after multiple learning steps. The network structure in which 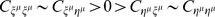 underlies the chaotic spontaneous activity with high closeness to the learned target patterns and successful recall of the target upon application of the corresponding input. At the border between the R and the NR regimes, 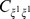 is much larger, while 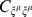 for 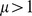 decreases rapidly with an increase in 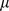. This network structure makes the approach of the spontaneous activity to the latest target (and reverse-target pattern) much closer as shown in Fig. 9C(i).

In the NR regime (i.e., with a much smaller 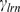 and much larger 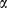), the decay rate of 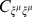 is much larger than that in the R regime and, only 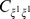 takes a significant value. For the latest mapping, the network structure is similar to that in the NR regime after one learning step as analyzed above. This is consistent with the existence of only the latest target and reverse-target attractors in the spontaneous activity and the absence of response to any input.

## Discussion

We have proposed an associative memory model with a simple learning rule that realizes the viewpoint of memories-as-bifurcations in which neural activities are transformed appropriately by each input to generate the requested targets. Using this viewpoint, we have analyzed the spontaneous activity and its response to a memorized input. With a Hebbian-type synaptic change based on the correlations between the pre- and postsynaptic neurons, the model succeeds in memorizing I/O mappings by sequential learning without losing earlier memories up to a capacity of 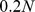 for a sufficient large 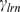 and small 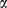. In the absence of input, the neural activity typically shows chaotic dynamics, while approaches to memorized target patterns are repeated from time to time. Upon inputs with adequate strength, e.g., the same as that used in the learning, flow structure of the neural activity is changed and the neural activity evolves into an attractor that matches the requested target pattern corresponding to each input. The neural activity dynamics change from spontaneous activity with a large variability in a high-dimensional state space to a lower-dimensional state that loses the variability with an increase in the input strength, which we understand as successive bifurcations. Interestingly, the synaptic connections generated by learning share a common property as those that were previously designed based on correlations in the input and target patterns [34]. We outline here the significance of our viewpoint and the consequences of our results for neuroscience.

### Memories as a result of sequential learning

We introduced a sequential learning rule, to match the memories as bifurcations viewpoint, by adopting a simple rule based on the correlations between the activities of the pre- and postsynaptic neurons; the rule is similar to the perceptron learning rule [13], [16].

Sequential learning or palimpsest learning have been studied over a few decades [38]–[40], [42], [43], and it has been shown that learning a new I/O mapping can easily destroy traces of previously memorized target patterns to such an extent that the memory capacity is lower than that for non-sequential learning. Methods have been proposed to alleviate the decrease in the capacity by decreasing the degree of synaptic plasticity, for example, by decreasing the number of the synapses that change simultaneously [42]. However, the destruction of earlier attractors due to the formation of new attractors is still a general trend as long as the memorized targets are attractors in the same dynamical system.

From our viewpoint, in contrast, the attraction to a new learned target is shaped under a “different” dynamical system because each system exists in the presence of a different input pattern, and as we demonstrated, the neural network does not completely lose the memory learned earlier; the capacity is 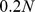 for an input strength 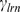 that is sufficiently large and a rate of synaptic change 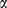 that is sufficiently smaller than that of the change in the neural activity. For larger 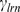 values, the system under the new input deviates farther from that without input and from that with the previously learned inputs so that the traces of the previously learned memories are not destroyed. For small 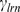, in contrast, the system under the input is close to that without input, so that the traces are easily destroyed. For a larger 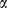, on the other hand, the change in the synaptic connection is larger so that traces of previously learned memories are destroyed, while the synaptic connection is enhanced and selectively stabilizes the new target pattern. Indeed, for a larger 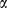 and smaller 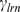 area, only a highly stable attractor that matches the latest target is generated by removing earlier memories, and thus multiple mappings are not memorized.

The simplicity of our learning scheme may have potential applications for the learning algorithm of I/O mappings. A limitation in our model is that the target information is supplied to all neurons because we used all-to-all recurrent connections. This limitation can be overcome by appropriately introducing a layered network structure and reinforcement learning algorithm [33] into the present learning algorithm. In addition, the present scheme is based on Hebbian-type synaptic changes that use only the pre- and postsynaptic neural activities and the target information under the presence of input; this means it may be plausible to expect the existence of such synaptic dynamics in biological neural system.

### Spontaneous activity and bifurcation into evoked activity

There have been extensive experimental studies on the responses of neural activities to external stimuli in the sensory cortex [1]–[4] and higher cortex area [5], [6]. Pre-stimuli, spontaneous activity had been dismissed as a background noise in these studies, but in recent experimental studies, it has been demonstrated that spontaneous neural activity without sensory input is not simple noise but is in fact highly structured in time and space [17], [44]. In particular, spontaneous activity is often found to exhibit transitory behavior among several activity patterns that are similar to those evoked by external stimuli [32], [35], [37], [45], [46]. In other words, spontaneous activity includes some patterns evoked by external stimuli [18]. Thus, spontaneous activity that is widespread and wanders over many patterns converges to one patterns by applying an input. If one observes a discontinuous change in the neural activity by increasing the input strength, we expect that the change will be interpreted as a bifurcation.

In the present study, we analyzed the transformation of the spontaneous to evoked activity from the memories-as-bifurcations viewpoint; we found that spontaneous activity that is chaotic but that often approaches the memorized targets is shaped by learning. This is reminiscent of the similarity between the spontaneous and evoked activities noted in the above experimental studies. Interestingly, if the spontaneous activity makes a closer approach to some target patterns, the inputs corresponding to those targets generate a higher neural activity response. This correlation between the responsiveness to a given input and the spontaneous activity may suggest a possible role of the spontaneous activity in preparing the response to the input.

There have been several studies of neural-network models of the spontaneous activity in neural dynamics in random networks or models of working memory [24]–[27]. Spontaneous activities that visit several patterns have been investigated as chaotic itinerancy over patterns [47], [48] or heteroclinic channels [49]. Our focus here lies in understanding whether such structure can be shaped by a simple learning rule and elucidating the characteristic behavior of the shaped spontaneous activity. Thus, our findings can also shed some light on how such transitory neural dynamics are generated.

We should note that, as an alternative approach contesting the memories-as-attractors viewpoint, the so-called liquid state machine was proposed [12], [50], [51], where learning I/O mapping was also achieved without multiple attractors. In this machine, there is a “reservoir” that stores the trace of the input and a “read-out unit” that detects this trace and transfers it to the desired output, while learning modifies only the read-out unit to generate the desired output. In our study, in contrast, there is no read-out unit, but the internal neural-activity dynamics (which corresponds to the reservoir) is modified during the learning process. With this approach, we can study spontaneous neural activity dynamics and evoked activity dynamics, which are not considered in the liquid-state machine.

### Simple learning rule can shape the spontaneous activity wandering among the memorized targets

A recent study by Berkes et al., [32] has demonstrated that the similarity between the spontaneous and evoked neural activities is not an innate property but is shaped through a developmental process; the dynamics of the activities are expected to be modified by the experience-dependent synaptic plasticity, and as a result, the similarity is believed to be shaped. We have shown that such a similarity is shaped through sequential Hebbian learning. In addition, we have found that in the network connection matrix, the characteristic pattern of the matrix elements (Eq. 7) is also shaped, although the learning rule can form another characteristic pattern of network connections. In a parameter regime without any memory capacity, only the 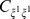 element is significant. In striking contrast, in a regime with memory capacity of I
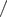
O mappings, the values of the elements of 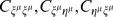, and 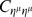 are of a comparable order, with the former two being positive and the latter two being negative. This network structure (the sign of each element) is found to be in common with the network in [34], which was designed to achieve appropriate bifurcations upon certain inputs by superposing connections generated by the correlation between each target and input pattern with equal weight. In the present study, such connections, even though the weights are biased to recently memorized patterns, are generated as a result of a simple learning rule. This demonstrates the generality of the memories-as-bifurcation viewpoint and the existence of a variety of connections for its implementation.

### Biological plausibility of the synaptic dynamics

Finally, we briefly discuss the biological plausibility of our learning rule. Indeed, it does not follow the Hebbian unsupervised learning adopted in standard models for the cerebrum cortex with recurrent neural connections. Still, our learning rule also satisfies a minimum requirement for a biological neural system [42]: a learning rule needs only local information for pre- and postsynaptic cells and does not require any global information, which is difficult for each neuron to obtain. In fact, our learning model given by Eq. 2 needs information on only the neural activity of the pre- and postsynaptic cells and the target activity in the postsynaptic cell.

The learning rule (Eq. 2) consists of two parts: an anti-Hebbian part [52], [53], 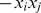, and the supervised part, 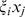. First, a possible interpretation of the anti-Hebbain rule can be provided by introducing an interneuron. It is known that the excitatory neurons (pyramidal neurons) are connected through inhibitory neurons (interneurons) in the sensory cortex. When activations of pre- and post excitatory neurons are correlated and synapses between the presynaptic excitatory neuron and the inhibitory interneuron and those between the interneuron and the postsynaptic neuron are strengthened by the Hebbian rule, the efficacy between the pre- and postsynaptic neurons is effectively weakened. Instead of taking into account these intermediate neurons explicitly, one could eliminate variables for the interneurons and consider effective direct coupling between 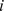 and 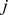, as in our model. In this case, the coupling between 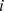 and 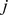 follows anti-Hebbian plasticity of the synapse.

To discuss the plausibility of the supervised part, let us consider another network whose activity represents target pattern 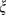 and which projects onto the network in our model. Here, the target pattern does not represent a signal to error of the output behavior, as often used in supervised learning models in the cerebellum cortex [54], [55], but represents only the neural activity pattern to be learned. The term 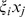 represents a simple Hebbian change between the presynaptic neurons in the network and the other network representing the target. This Hebbian change enables learning the correlation between the activities in the target network and in the presynaptic neurons. This example is only one possible way to implement our model in a biological neural network, and future studies are needed to establish a link between our learning rule and more biological neural-network dynamics.

## Supporting Information

Figure S1
**Overlap with the target and the input patterns after 40 learning steps in the R regime.** **A.** The average overlap 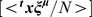 in the presence of the 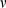-th input as a function of 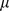 and 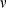. **B.** The average overlap 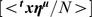 in the presence of the 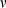-th input as a function of 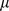 and 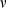. We used the same parameters as those in Fig. 7 and computed the overlap by averaging over 100 network and 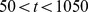. One can find that, upon application of an input 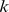, the overlap with the requested target 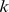 is selectively higher than the other overlaps.(EPS)

Figure S2
**The neural dynamics after 40 learning steps in the non-response (NR) regime.** **A.** The time series of the neural activities shown by the overlap with the 1st, 5th, and 30th targets 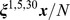 in the absence and presence of the 1st (red), 5th (green), and 30th (blue) inputs (shown by the colored bars above the plot) for 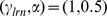. **B.** The time-averaged overlaps with the learned targets as a function of 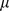 (squares). The time- and ensemble-averaged overlaps with the targets and inputs are shown as the solid and dashed lines, respectively. **C.** The average overlap 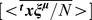 in the presence of the 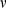-th input as a function of 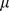 and 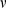. **D.** The average overlap 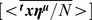 in the presence of the 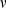-th input as a function of 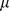 and 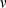. In all figures, we used the time series 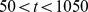 as the time-averaged overlap and the ensemble-averaged one.(EPS)

Figure S3
**Dependence of evoked neural activity on the number of the elements.** The overlap of evoked activity with 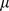-th target for different 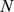 is shown as a function of 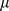 divided by 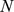. The curves for 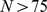 converge to a unique curve, by scaling the index 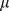 of the learned mappings divided by 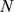. We computed the overlaps by averaging over time 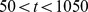 for all 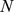 and over 100 networks for 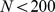 and 50 networks for 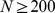.(EPS)
